# Targeting Glioblastoma Stem Cells: A40s Aptamer-NIR-Dye Conjugate for Glioblastoma Visualization and Treatment

**DOI:** 10.3390/biom15060768

**Published:** 2025-05-27

**Authors:** Alessandra Affinito, Francesco Ingenito, Sara Verde, Emanuele Musella, Birlipta Pattanayak, Danilo Fiore, Cristina Quintavalle, Aurelia Fraticelli, Martina Mascolo, Gianluca Petrillo, Claudia Pignataro, Giada De Luca, Laura Mezzanotte, Gerolama Condorelli

**Affiliations:** 1 Department of Molecular Medicine and Medical Biotechnology, University of Naples Federico II, 80131 Naples, Italy; alessandra.affinito@unina.it (A.A.); francesco.ingenito@outlook.it (F.I.); emanuele.musella@outlook.com (E.M.); birlipta.pattanayak@gmail.com (B.P.); danilo.fiore@unina.it (D.F.); martina.mascolo@outlook.com (M.M.); gianlucapetrillo.gp@gmail.com (G.P.); claudia-pignataro@libero.it (C.P.); giada.deluc@gmail.com (G.D.L.); 2 AKA Biotech, 80131 Naples, Italy; saraverde98@outlook.com (S.V.); aureliafraticelli98@gmail.com (A.F.); 3 Department of Biomedicine and Prevention, University of Rome “Tor Vergata”, 00133 Rome, Italy; 4 Institute of Endotypes in Oncology, Metabolism and Immunology “Gaetano Salvatore” (IEOMI) CNR, 80131 Naples, Italy; cristina.quintavalle@cnr.it; 5 Department of Molecular Genetics, Erasmus MC Cancer Institute, University Medical Center Rotterdam, 3015 CE Rotterdam, The Netherlands; l.mezzanotte@erasmusmc.nl

**Keywords:** GBM, aptamers, A40s, NIR dye, glioblastoma stem cells, photodynamic therapy, PDT, ICG, IR700DX, EphA2, guide surgery

## Abstract

Glioblastoma (GBM) is the most aggressive and challenging brain cancer, in terms of diagnosis and therapy. The highly infiltrative glioblastoma stem cells (GSCs) are difficult to visualize and surgically remove with the current diagnostic tools, which often lead to misdiagnosis and false-positive results. In this study, we focused on a groundbreaking tool for specifically visualizing and removing GSCs. We exploited the specific binding of A40s aptamer to EphA2 for the selective delivery of Near-Infrared Dyes (NIR-Dyes), like IR700DX and ICG, both in vitro and in vivo. The A40s aptamer, engineered through the NIR-Dye conjugation, did not affect aptamer binding ability; indeed, A40s-NIR-Dye conjugates bound GLI261 stem-like cells and patient-derived GSCs in vitro; moreover, they induced cell death upon photodynamic therapy treatment (PDT). Additionally, when systemically administrated, the A40s-NIR-Dye conjugates allowed GSC visualization and accumulated in tumor mass. This allows GSCs detection and treatment. Our findings demonstrate the potential use of A40s aptamer as a targeted therapeutic approach and imaging tool in vivo for GSCs, paving the way for improved, more effective, and less invasive GBM management.

## 1. Introduction

Glioblastoma multiforme (GBM), also known as grade IV astrocytoma, represents the most aggressive and lethal brain tumor in adults with a dismal medical outcome and a median overall survival of only 15 months [1]. It arises from glial cells or their progenitors, and treatment modalities are currently limited to surgical resection followed by radiation therapy accompanied by the administration of an alkylating chemotherapy drug, temozolomide (TMZ). GBM is characterized by high molecular heterogeneity. In this regard, a small GBM cell population, named Glioma Stem Cells (GSCs), is responsible of the development, growth, and maintenance of GBM, expressing high resistance to conventional treatments [2]. Phenotypically, GSCs are highly invasive and often colonize the surrounding healthy tissue, hampering the complete surgical removal of the mass [3,4]. The Eph receptor tyrosine kinase (EphA2) is a GSCs marker essential for GSCs pool maintenance, self-renewal, and tumorigenesis, ultimately promoting GBM proliferation and invasiveness [5,6,7,8]. Therefore, GSCs, present as residual disease after primary debulking surgery and conventional therapies, currently represent the main reason for GBM relapse [9,10].

An additional challenge in GBM management consists in the difficulty diagnosing and monitoring the disease along treatments. Indeed, histopathological analyses are limited by low accessibility to the tumor mass, while imaging approaches (magnetic resonance imaging, MRI, which is the gold standard, and computed tomography—CT) present several limitations [11]. Indeed, while CT offers less detailed images, MRI is not able to distinguish GBM stages, residual or recurrent tumor changes, or other mass lesion types. Additionally, even though the use of FDA-approved intraoperative fluorescent agents, near-infrared dyes (NIR-Dyes), indocyanine green (ICG) and IR700DX, or the agent 5-aminolevulinic acid (5-ALA) is increasingly applied to surgically maximize the resection of malignant gliomas, these fluorescent dyes are not tumor-specific, causing a low predictive value of the technique [12,13,14,15]. In particular, ICG is an amphiphilic cyanine dye with absorption and emission peaks at 789 nm and 813 nm, respectively, and has been used in medical imaging since the mid-1950s [Quest Graph™ Spectrum [ICG (Indocyanine green)].” AAT Bioquest, Inc., Pleasanton, CA, USA, 27 March 2025) [16]. In contrast, IR700DX is a phthalocyanine dye with an excitation peak at 689 nm and an emission peak at 699 nm, currently under investigation as photoimmune conjugate in various clinical trials, including a post-marketing clinical trial (NCT02422979), (Quest Graph™ Spectrum [IRDye 700DX].” AAT Bioquest, Inc., Pleasanton, CA, USA, 27 March 2025). Both dyes exhibit non-specific binding to proteins, leading to non-specific targeting. However, they exert therapeutic effects through several mechanisms, such as ROS generation, induction of oxidative stress, and cell damage, which lead to cell death [17,18,19,20]. As consequence, despite their use improving progression-free survival and overall survival of GBM patients, both instrumental analysis and guided imaging can result in misdiagnosis and false-positive and negative results [21,22,23].

Nanotechnology demonstrates significant potential for therapeutic and delivery techniques; in this scenario, nanoscale synthetic RNAs molecules named aptamers have high importance [24,25]. Aptamers are short three-dimensional structures of single-stranded nucleic acids (RNA or DNA), which are in some cases able to overcome the Blood–Brain Barrier (BBB) through several mechanisms, to recognize and bind the protein target with high selectivity and affinity; they are characterized by low immunogenicity and toxicity [26,27,28,29]. These molecules can be conjugated with fluorophores, radionuclides, and magnetic or contrast agents [30,31,32]. Furthermore, aptamers can also be used as delivery systems to transport therapeutic agents in target tissues; indeed, they can specifically deliver photosensitizers for the precise photodynamic therapy (PDT) of cancer [33].

In view of this, we previously selected and characterized a 30-mer aptamer, named A40s, able to specifically bind GSCs through direct recognition of the EphA2 receptor [34,35]. In our research, we developed a unique A40s-based fluorescent tool to specifically target GSCs. This aptamer has been conjugated to NIR-Dyes, with the aim of developing a sensitive approach to localize GBM in vivo and to induce GSCs death through PDT. Given the importance of implementing specific and minimally invasive therapies for targeting GSCs, along with the need to improve diagnosis and monitoring, this tool addresses current therapeutic limitations by enhancing GBM visualization in vivo and facilitating better tumor removal during surgery, finally improving the efficacy of GBM treatment.

## 2. Materials and Methods

### 2.1. Cell Culture

Patient-derived GBM cells were obtained from the Institute of Neurosurgery, School of Medicine, Università Cattolica, Rome, Italy, after craniotomy of adult patients from which, before surgery, informed consent was obtained. Mechanical dissociation of GBM tumor specimens allowed stem cell isolation, as previously described [35,36]. GSCs derived from patient specimens that were not immortalized were then cultured in a serum-free medium supplemented with EGF and fibroblast growth factor beta (bFGF) (Sigma-Aldrich, Milan, Italy). Differentiated GL261-luc2 cells (Perkin Elmer, Waltham, MA, USA) were cultured in Dulbecco’s modified Eagle medium (DMEM) supplemented with 10% FBS (Gibco Life Technologies, Monza, Italy) and antibiotics/antimycotics (100 U/mL penicillin and 100 μg/mL streptomycin, Gibco Life Technologies, Monza, Italy), while the stemness was induced by culturing them in DMEM/F12 with B27 supplement, EGF, and bFGF.

### 2.2. A40s Conjugation to NIR-Dyes

A mass of 25 ug of A40s or non-related aptamer (Scra) modified with an NH_2_ group at 3′end (CityofHope, Duarte, CA, USA) was resuspended in PBS 1 mg/mL and denatured–renatured at 85 °C for 5′, ice for 2′, and 37 °C for 10′. IRdye700DX-NHS (Licor Biosciences, Lincoln, NE, USA) and ICG-NHS (Pulsion Medical Systems, Feldkirchen, Germany) was purchased and manufacturers’ protocols applied to let the NHS ester react with amino groups of the modified aptamer to generate NIR-Dye chimera molecules. One-to-one molar ratio of dye was added for the conjugation in 1M potassium phosphate buffer pH 9. The mix was incubated for 3 h at 20 °C. Conjugates were then purified by a 75 µL Zeba spin column 7k (Thermo Scientific, Milan, Italy) with a buffer exchange procedure to eliminate free dyes; the ratio of aptamer/dye was quantified by simultaneously measuring the absorbance of the RNA (A_280_) and IRdye700DX (A_689_) with a nanodrop spectrophotometer ND-1000 and ICG Dye (A_800_) with TECAN infinite 200pro M plex; then, the conjugate was characterized by non-denaturing 12% acrylamide gel electrophoresis in TBE buffer and/or size-exclusion HPLC (SE-HPLC). The scrambled sequence of an unrelated aptamer was used as a negative control: 5′UUCGUACCGGGUAGGUUGGCUUGCACAUAGAACGUGUCA3′ [37]. A40s sequence is patented (WO2020230047A1). 

### 2.3. Aptamer Binding

A quantity of 2 × 10^5^ patient-derived GSCs or GL261-luc2 stem-like cells was seeded and treated with 200 nM of aptamers for 30 min at 37 °C in F12 free media in the presence of 100 μg/mL polyinosine (Sigma-Aldrich, Milan, Italy) used as a nonspecific competitor. Unbound RNA was removed by twice washing with PBS. Cells were lysed in TRIzol (Thermo Fisher Scientific) containing 0.5 pmol/mL of a non-related sequence used for normalization. Bound aptamer was quantified by performing Custom TaqMan Small RNA Assays (Thermo Fisher Scientific) following the manufacturer’s recommendations. Likewise, chimera binding was evaluated by fluorescence scanning (Odyssey CLx Imager, Lincoln, NE, USA). Here, 2 × 10^5^ of Gl261-luc2 stem-like cells were incubated with A40s-NIR-Dye chimera for 15 and 30 min, then fluorescence intensity was assessed (ex: 685 nm, em filter: 710–730 nm).

### 2.4. Cell Death and Cell Viability Quantification

A quantity of 2 × 10^3^ patient-derived GSCs or GL261-luc2 stem-like cells was seeded in 96-well plates in triplicate and incubated with 400 nM of A40s-NIR-dye chimera or controls (Scra-NIR-Dye or free NIR-Dye) for 15 min at 37 °C in the dark into a 5% CO_2_ incubator. Then, they were exposed to 10 J/cm^−2^ light via an NIR light-emitting diode (range emission 680–700 nm wavelength). Apoptosis and cell viability were analyzed after 48 h and 72 h with Caspase-Glo^®^ 3/7 Assay Systems (Promega, Milan, Italy) and CellTiter 96 AQueos One Solution Cell Proliferation Assay (MTS) (Promega, Milan, Italy), respectively, as previously described, according to the manufacturers’ protocols [38,39].

### 2.5. Protein Isolation and Western Blotting

PBS-washed cells were lysed in JS buffer (50 mM HEPES, pH 7.5, containing 150 mM NaCl, 1% Glycerol, 1% Triton X100, 1.5 mM MgCl_2_, 5 mM EGTA, 1 mM Na_3_VO_4_, and 1X protease inhibitor cocktail) for 45 min in ice, as previously described [40]. Proteins were quantified by the Bradford assay (BioRad, Milan, Italy), and equal amounts of proteins were separated by SDS-PAGE (10% acrylamide), then transferred onto nitrocellulose membranes (G&E Healthcare, Milan, Italy) and incubated at 4 °C overnight with primary antibodies after 1 h with 5% non-fat dry milk in Tris-buffered saline (TBS)-0.1% Tween-20 blocking; detection was performed using the enhanced chemiluminescence system (Thermo, Euroclone, Milan, Italy). The primary antibodies used were anti-EphA2 (Santa Cruz Biotechnology, Dallas, TX, USA) and anti-β actin (Sigma-Aldrich, Milan, Italy).

### 2.6. In Vivo and Ex Vivo Biodistribution

As a proof-of-concept experiment to qualitatively assess the biodistribution of the labeled aptamer, 2 × 10^6^ Gl-261-luc2-stem-like cells were subcutaneously engrafted in *BALB/C* nude mice flank (n = 3), following anesthesia with isoflurane (1.5%). After 21 days, ICG (Sigma Aldrich) (n = 1) and A40s-ICG (n = 2) conjugate were intravenously injected at a 2 nM equimolar dose in 100 microliters of PBS. For in vivo distribution evaluation, mice were scanned 1, 6, and 24 h after treatments were performed. Mice were sacrificed by cervical dislocation at 6 and 24 h after treatments, and liver, gut, kidneys, spleen, heart, and brain were collected and imaged ex vivo. The scanning was performed with an IVIS Spectrum imaging system (PerkinElmer, Waltham, MA, USA). The scanning parameters were as follows: excitation wavelength = 745 nm; emission wavelength = 820 nM; exposure time = 2 s; binning factor = Medium; f/Stop = 2; Field of View = D.3.

### 2.7. Statistical Analysis

Continuous variables are given as mean ± SD. Data were analyzed for significance with GraphPad Prism 8.0.2 (San Diego, CA, USA) using Student’s *t*-test (two variables) or one-way ANOVA (more than two variables). A *p* value ≤ 0.05 was considered significant for all analyses.

## 3. Results

### 3.1. A40s-Based Chimeras

To specifically target GSCs and enhance GBM imaging, we conjugated A40s aptamer with two NIR-Dyes, IR700DX and ICG. In detail, a modified A40s, bearing a terminal amino group (-NH2), was linked with the ester group of the dyes to be used for PDT and as an in vivo imaging tool, respectively.

**Figure 1:**
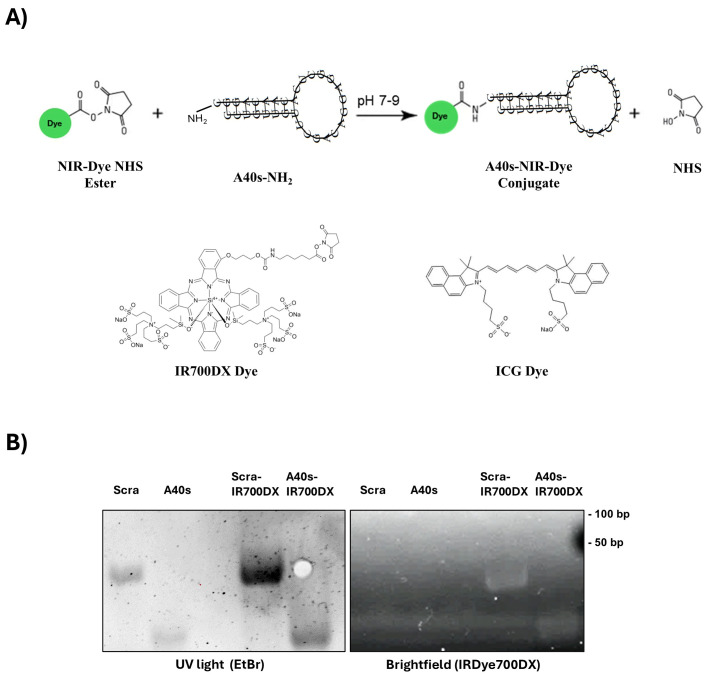
A40s-NIR-Dye conjugates characterization. (**A**) Schematic representation of the reaction and the resulting aptamer-NIR-Dye conjugate. (**B**) Non-denaturing 12% acrylamide gel electrophoresis of A40s-IR700DX chimera and controls (non-related aptamer-based chimera, Scra-IR700DX, and non-labeled aptamers); simultaneous visualizing of the dye portion by white light irradiation (right panel) and RNA by ultraviolet (UV) light (left panel). Original images can be found in Supplementary Materials.

A one-to-one molar ratio of aptamer and dye was used to generate chimeric conjugates according the schematic representation in Figure 1A, and the proper generation was checked by simultaneous visualization of RNA and NIR-Dye through non-denaturing gel electrophoresis. Due to the small size of the dye, no clear shift in molecular weight was visible on the gel. However, in both chimeras, the conjugates exhibited a visible dye signal, which is absent in the unconjugated aptamers and localized at the aptamer band level (Figure 1B). To further corroborate the A40s-ICG linkage, we executed a size-exclusion high-performance liquid chromatography (SE-HPLC) analysis. The matching retention time for the nucleic acid and ICG, obtained with HPLC paired with both diode array (DAD) and fluorescence (FLD) detection, confirmed the effective conjugation of the aptamer to the dye (Supplementary Figure S1A). These findings proved the feasibility to label A40s aptamer with NIR-Dyes.

### 3.2. A40s-NIR-Dye Binding to GSCs

**Figure 2:**
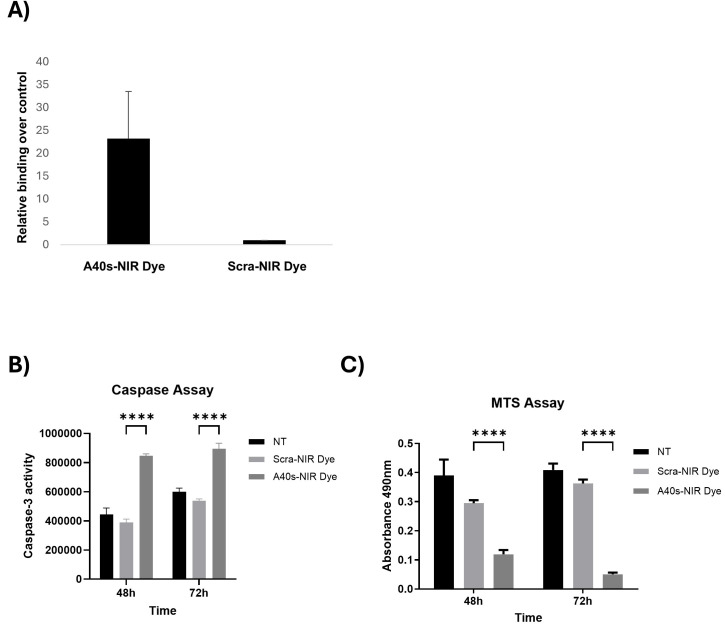
Binding ability and functional assay of A40s-IR700DX on GSCs. (**A**) Binding ability of A40s-IR700DX chimera on patient-derived GSCs. Representative experiment is shown, and result is expressed relative to the background binding detected with a scrambled chimera used as a negative control. (**B**) Caspase activity and (**C**) cell viability of patient-derived GSCs 48 h and 72 h after 400 nM A40s-IR700DX or Scra-IR700DX chimera treatment. Results are presented as mean ± SD of three replicates. **** *p* ≤ 0.0001.

The ability of A40s to recognize patient-derived GSCs has been previously demonstrated [35]. However, since NIR-Dye could affect aptamer shape, we checked the preservation of the binding ability of the newly synthesized chimeras. Here, 200 nM A40s- or Scra-NIR-Dye conjugate was used to treat patient-derived GSCs, and their binding ability was checked by RT-qPCR, as previously described [35]. As indicated in Figure 2A, A40s retained its capability to bind GSCs over the control, even after conjugation with the IR700DX NIR-Dye. Further, we tested the functional effects of the chimera. To this aim, GSCs were treated with 400 nM of A40s-NIR-Dye chimera or a non-related aptamer-based chimera (Scra-NIR-Dye) and subsequently activated by light irradiation (10 J/cm^2^) to induce phototoxicity of the conjugate, in order to test its ability to induce cell death and reduce cell viability. To this purpose, we assessed caspase-3 activation and performed an MTS assay after 48 h and 72 h of treatment. The A40s-IR700DX chimera induced apoptotic cell death and led to the reduction of cell viability compared to controls (Figure 2B,C). These data suggest that A40s-NIR-Dye chimera has potential effects in targeting GSCs.

### 3.3. A40s-NIR-Dye PDT on GSCs

**Figure 3:**
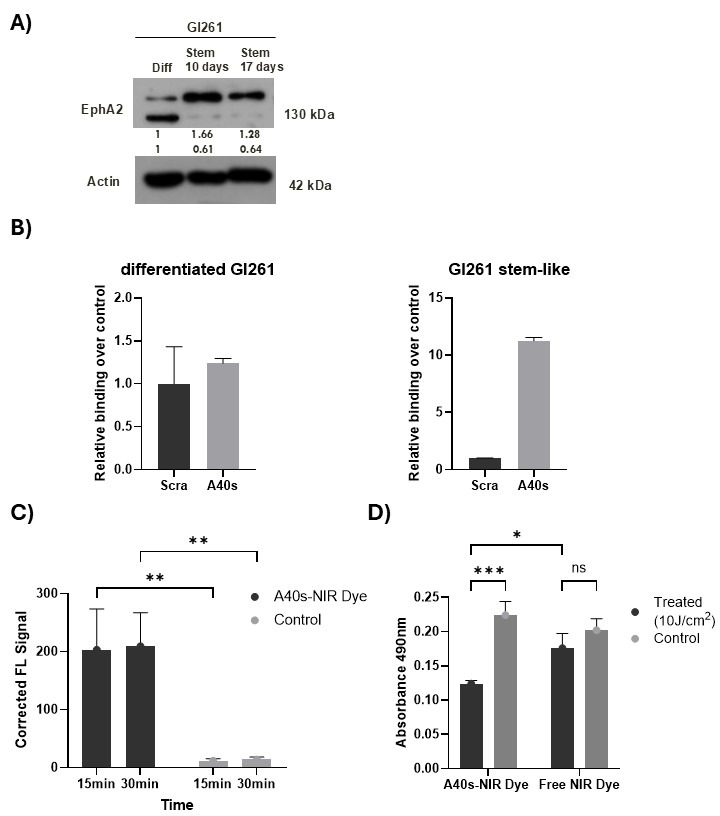
GL261-luc2- stem-like cells as a GBM mouse model. (**A**) Western blot analysis of EphA2 expression in GL261 differentiated and stem-like cells (after 10 and 17 days). (**B**) A40s aptamer binding ability on differentiated and GL261-luc2 stem-like cells. Representative experiment is shown, and result is expressed ± SD relative to the background binding detected with a scrambled chimera used as negative control. (**C**) A40s-IR700DX chimera binding ability after 15 and 30 min of incubation with GL261 stem-like cells evaluated by fluorescence scanning (Odyssey CLx Imager). Representative experiment is shown, and result is expressed ± SD relative to the background binding detected with a scrambled chimera used as negative control. (**D**) Cell viability of GL261 stem-like cells (MTS assay) upon 10 J/cm^2^ after 1 h treatment with A40s-IR700DX chimera and NIR-dye free. Results are presented as mean ± SD of three replicates. * *p* ≤ 0.05; ** *p* ≤ 0.01; *** *p* ≤ 0.001. Original images can be found in Supplementary Materials.

To study the imaging applicability of the generated chimeras, we took advantage of a syngeneic mouse model of GBM, based on GL261-luc2 cells. Therefore, GL261-luc2 differentiated cells were first cultured in suspension as stem-like cells (3D culture—Supplementary Figure S1B). Afterwards, we evaluated stem cell markers, including EphA2, targeted by the A40s aptamer. Western blot analysis demonstrated EphA2 upregulation in GL261-luc2 cells cultured for 10 and 17 days in stem-like condition (Figure 3A). Then, we assessed the binding capability of A40s and A40s-chimera. In accordance with previous data, A40s discriminated stem-like cells from their differentiated counterpart (Figure 3B); likewise, A40s-NIRdye chimera was able to bind GL261-luc2 stem-like cells upon 15 min of treatment (Figure 3C). Moreover, A40s-NIRdye treatment proved to reduce GL261-luc2 stem-like cell viability (Figure 3D), demonstrating the phototoxic potential of the conjugate on GL261-luc2 stem-like cells. 

### 3.4. In Vivo Biodistribution of A40s-NIR-Dye and Ex Vivo Evaluation

**Figure 4:**
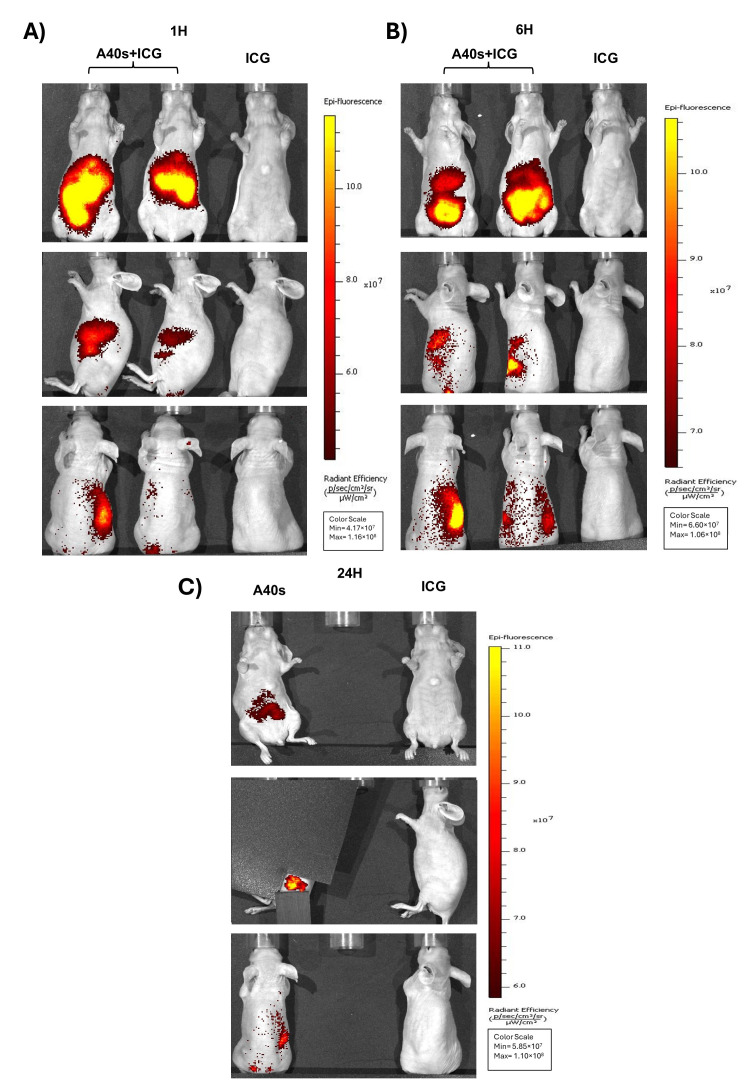
A40s-ICG chimera in vivo biodistribution on GL261-luc2 stem-like cells engrafted into *BALB\C* nude mice. A40s-ICG chimera and ICG free clearance evaluation after 1 h (**A**), 6 h (**B**), and 24 h (**C**) treatments by IVIS Spectrum imaging system.

**Figure 5:**
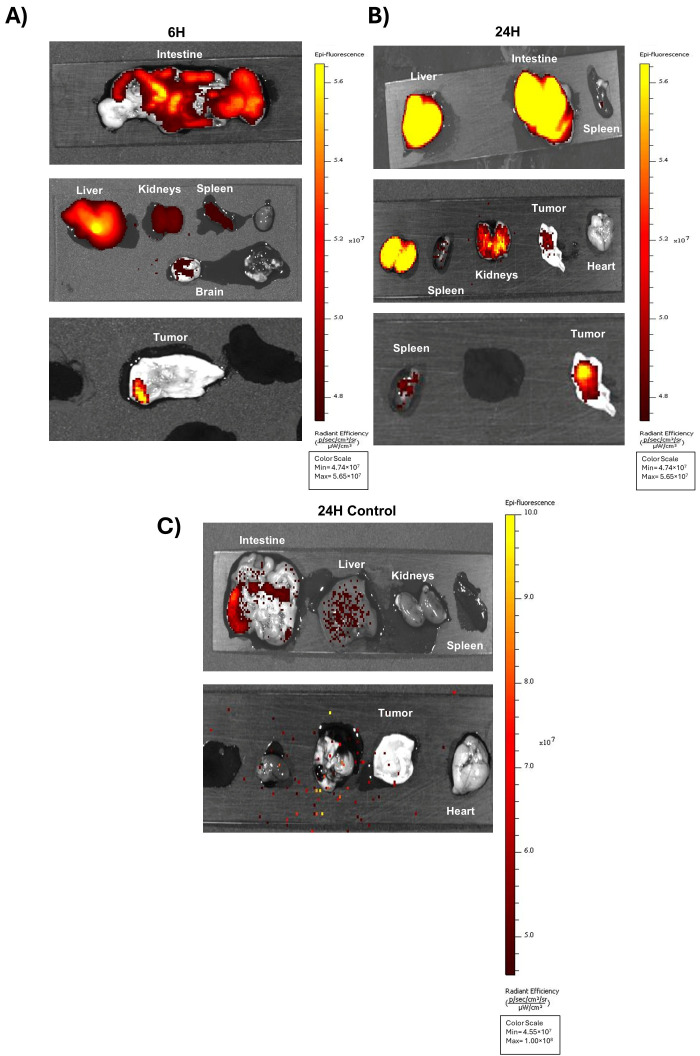
A40s-ICG chimera ex vivo biodistribution in GL261-luc2 stem-like cells engrafted into *BALB\C* nude mice. A40-ICG ex vivo detection in organs by IVIS Spectrum imaging system after 6 h (**A**) and 24 h (**B**) treatments; (**C**) organs of control mouse treated with free ICG after 24 h of treatment.

We assessed the A40s-based chimera in an in vivo mouse model. Given the favorable NIR emission of ICG for Real-Time Fluorescence Imaging with its excellent tissue penetration and low background signal [41,42,43,44], A40s-ICG chimera was generated as mentioned above and used in in vivo experiments, with the main purpose of evaluating chimera biodistribution. GL261-luc2 stem-like cells were subcutaneously engrafted in both the flanks of *BALB/C* nude mice and monitored until the tumor mass became palpable. Subsequently, mice were intravenously administered the A40s-ICG chimera or the Free ICG, with the latter acting as a negative control. Afterwards, the biodistribution profile of the two administered formulations was followed by fluorescence over time. Imaging acquisition showed a rapid clearance of the free ICG, in accordance with the few minutes of ICG half-life [45]. Indeed, ICG was quickly eliminated by the intestines, as demonstrated by low-intensity fluorescence within one hour after administration (Figure 4A). Contrarily, the aptamer crucially altered the NIR-Dye distribution to tissues by reducing its clearance. Indeed, A40s-ICG chimera after 1 h of treatment was still detectable in vivo. The conjugate appeared partially distributed in the intestine and stomach, partially sharing the ICG distribution and its elimination into the bile; however, it was eliminated also by the kidneys, according to the aptamer excretion (Figure 4A) [46,47]. Six hours after the injection, the signal related to the chimera was still present with a distal progression in the intestine and with a slight increase in the kidneys (Figure 4B and Figure 5A). At twenty-four hours, the general signal intensity was reduced to about 80% (Figure 4C, and Supplementary Figure S1C) but a slight increase in the kidneys and tumor was notable (Figure 5B, and Supplementary Figure S1D), demonstrating the partial renal elimination of the compound and its accumulation in the tumor mass. On the contrary, no accumulation of the chimera was observed in the heart. Moreover, 24 h after treatment, ICG was rarely present in the intestine and liver, and no signal was detectable in the tumor, further substantiating the ability of A40s to deliver the NIR-Dye to the target cells (Figure 5C).

Our findings proved the potential use of A40s aptamer as a targeted therapeutic and imaging tool for GSCs, opening the way to new potential therapeutic and diagnostic applications for GBM.

## 4. Discussion

GBM is the most aggressive brain tumor and poses many challenges in terms of diagnosis and therapy. Characterized by high invasiveness, molecular heterogeneity, and rate of relapse, GBM treatment has not improved in the last 20 years in clinical management. Despite important research efforts, treatments are still limited to alkylating agents and radiotherapy; no significant advances have been made in overall survival, and the prognosis of GBM patients remains very poor [48,49]. Recurrence is almost inevitable due to infiltrative cells, which are difficult to visualize and surgically remove.

It has been demonstrated that the extent of resection is a crucial factor for patients’ outcomes, positively correlating with overall survival. However, conventional imaging often fails to accurately outline GBM masses. This unmet medical need results in many current clinical trials that aim to improve tumor resection (NCT05399524-NCT02795364) and visualization (NCT04588454-NCT01252459-NCT03903419) in order to enhance GBM management.

Several tracers have been approved by the FDA so far to treat cancer, such as ICG, IR700DX, and 5-ALA, to be used alone or vehiculated by antibodies.

In this study, we focused on a groundbreaking method to simultaneously and specifically visualize and remove the most infiltrative GBM cells, GSCs, through the specific binding of A40s to EphA2 receptors. This innovative tool, composed of A40s and a NIR-Dye, on one hand selectively recognizes the GSCs and, on the other hand, allows their visualization and leads to their death through PDT. We first proved in vitro the abilities of the conjugate in binding GSCs, then we evaluated the potential in inducing cell death. The newly generated chimera proved to recognize GSCs and to reduce cell viability by inducing cell apoptosis. Therefore, we studied the clinical applicability of the conjugate in vivo. As shown, the chimera was systemically distributed after injection, and it strongly prolonged NIR-Dye retention, letting it accumulate in the tumor mass. Notably, although the conjugate also accumulated in the intestine (following the typical biodistribution of ICG), it is important to point out that while the free dye was rapidly eliminated, the chimera persisted in the mice. This indicates that the aptamer affects NIR-Dye accumulation and elimination. Thanks to A40s, the conjugate is accumulated in the tumor mass over time, thus potentially enhancing the diagnostic and therapeutic efficacy of the dye. Furthermore, the intestinal accumulation is compatible with the primary goal of the conjugate, brain tumor visualization, as well as its potential use as a therapeutic agent.

Indeed, the new generated conjugate can exploit the NIR-Dye-mediated PDT advantages, like the low invasiveness compared to surgery, the possibility to repeat the treatment as opposed to radiotherapy, and a more accessible cost compared to other cancer treatments. Nevertheless, NIR-Dyes present some limitations, the main one being the limited NIR light penetration (~1 cm), which restricts their applicability to accessible tumors like cutaneous cancers [50,51]. What we propose here, with a series of proof-of-concept experiments, is to exploit A40s-based conjugates for delivering photosensitizers, and thus improve tumor visualization, and, in parallel, enable better removal of GSCs during surgery (through cell visualization or PDT approaches). Even considering the exploratory nature of this study and the need for future research to fully exploit these findings, particularly regarding the therapeutic potential of the conjugates, this study paves the way for more successful diagnostic and therapeutic strategies for GBM, intending to revolutionize GBM clinical approaches.

## 5. Conclusions

In conclusion, GBM remains a highly aggressive and difficult-to-treat brain tumor, with limited advancements in patient survival despite ongoing research efforts. Current treatments, while somewhat effective, are hindered by challenges in tumor visualization, incomplete resection, and the highly infiltrative nature of the tumor cells, particularly GSCs. This study highlights an innovative approach utilizing A40s-based conjugates to target EphA2 receptors on GSCs, offering both diagnostic and therapeutic potential. By leveraging NIR-Dye-mediated PDT, this method provides a promising alternative to traditional surgical and radiotherapy techniques, with the added benefit of minimizing invasiveness and recurrence. Although there are limitations to overcome, particularly related to NIR light penetration, this research opens new avenues for improving GBM management, suggesting that future developments in this area could lead to more effective, less invasive treatments for patients.

## Data Availability

Data produced in the current study are available from the corresponding author upon reasonable request.

## Supplementary Materials

The following supporting information can be downloaded at: https://www.mdpi.com/article/10.3390/biom15060768/s1, Figure S1: A40s-ICG qualitative and in vivo quantitative evaluation.

## Abbreviations

The following abbreviations are used in this manuscript:

GBM	Glioblastoma
GSCs	Glioblastoma Stem Cells
NIR	Near-Infrared
PDT	Photodynamic Therapy
ICG	Indocyanine Green
BBB	Blood–Brain Barrier

## References

[1] Sabouri M., Dogonchi A.F., Shafiei M., Tehrani D.S. (2024). Survival rate of patient with glioblastoma: A population-based study. Egypt. J. Neurosurg..

[2] Eckerdt F., Platanias L.C. (2023). Emerging Role of Glioma Stem Cells in Mechanisms of Therapy Resistance. Cancers.

[3] Seker-Polat F., Pinarbasi Degirmenci N., Solaroglu I., Bagci-Onder T. (2022). Tumor Cell Infiltration into the Brain in Glioblastoma: From Mechanisms to Clinical Perspectives. Cancers.

[4] Vollmann-Zwerenz A., Leidgens V., Feliciello G., Klein C.A., Hau P. (2020). Tumor Cell Invasion in Glioblastoma. Int. J. Mol. Sci..

[5] Wykosky J., Gibo D.M., Stanton C., Debinski W. (2005). EphA2 as a novel molecular marker and target in glioblastoma multiforme. Mol. Cancer Res..

[6] Miao H., Gale N.W., Guo H., Qian J., Petty A., Kaspar J., Murphy A.J., Valenzuela D.M., Yancopoulos G., Hambardzumyan D. (2015). EphA2 promotes infiltrative invasion of glioma stem cells in vivo through cross-talk with Akt and regulates stem cell properties. Oncogene.

[7] Binda E., Visioli A., Giani F., Lamorte G., Copetti M., Pitter K.L., Huse J.T., Cajola L., Zanetti N., DiMeco F. (2012). The EphA2 receptor drives self-renewal and tumorigenicity in stem-like tumor-propagating cells from human glioblastomas. Cancer Cell.

[8] Qazi M.A., Vora P., Venugopal C., Adams J., Singh M., Hu A., Gorelik M., Subapanditha M.K., Savage N., Yang J. (2018). Cotargeting Ephrin Receptor Tyrosine Kinases A2 and A3 in Cancer Stem Cells Reduces Growth of Recurrent Glioblastoma. Cancer Res..

[9] Osuka S., Van Meir E.G. (2017). Overcoming therapeutic resistance in glioblastoma: The way forward. J. Clin. Investig..

[10] Garnier D., Meehan B., Kislinger T., Daniel P., Sinha A., Abdulkarim B., Nakano I., Rak J. (2018). Divergent evolution of temozolomide resistance in glioblastoma stem cells is reflected in extracellular vesicles and coupled with radiosensitization. Neuro-Oncology.

[11] Kwiatkowska-Miernik A., Mruk B., Sklinda K., Zaczynski A., Walecki J. (2023). Radiomics in the diagnosis of glioblastoma. Pol. J. Radiol..

[12] Stummer W., Pichlmeier U., Meinel T., Wiestler O.D., Zanella F., Reulen H.J., ALA-Glioma Study Group (2006). Fluorescence-guided surgery with 5-aminolevulinic acid for resection of malignant glioma: A randomised controlled multicentre phase III trial. Lancet Oncol..

[13] Aldave G., Tejada S., Pay E., Marigil M., Bejarano B., Idoate M.A., Diez-Valle R. (2013). Prognostic value of residual fluorescent tissue in glioblastoma patients after gross total resection in 5-aminolevulinic Acid-guided surgery. Neurosurgery.

[14] Mazurek M., Kulesza B., Stoma F., Osuchowski J., Mandziuk S., Rola R. (2020). Characteristics of Fluorescent Intraoperative Dyes Helpful in Gross Total Resection of High-Grade Gliomas-A Systematic Review. Diagnostics.

[15] Burley T.A., Maczynska J., Shah A., Szopa W., Harrington K.J., Boult J.K.R., Mrozek-Wilczkiewicz A., Vinci M., Bamber J.C., Kaspera W. (2018). Near-infrared photoimmunotherapy targeting EGFR-Shedding new light on glioblastoma treatment. Int. J. Cancer.

[16] Szpunar M., Aebisher D., Wal A. (2025). Changes in absorption spectra of indocyanine green after visible light exposure and cold dark storage. Spectrochim. Acta A Mol. Biomol. Spectrosc..

[17] Kishimoto S., Bernardo M., Saito K., Koyasu S., Mitchell J.B., Choyke P.L., Krishna M.C. (2015). Evaluation of oxygen dependence on and cytotoxicity of photoimmunotherapy using IR-700-antibody conjugates. Free Radic. Bio. Med..

[18] Kernt M., Hirneiss C., Wolf A., Liegl R., Rueping J., Neubauer A., Alge C., Ulbig M., Gandorfer A., Kampik A. (2012). Indocyanine green increases light-induced oxidative stress, senescence, and matrix metalloproteinases 1 and 3 in human RPE cells. Acta Ophthalmol..

[19] Tseng H.C., Kuo C.Y., Liao W.T., Chou T.S., Hsiao J.K. (2022). Indocyanine green as a near-infrared theranostic agent for ferroptosis and apoptosis-based, photothermal, and photodynamic cancer therapy. Front. Mol. Biosci..

[20] Smeets E.M.M., Dorst D.N., Franssen G.M., van Essen M.S., Frielink C., Stommel M.W.J., Trajkovic-Arsic M., Cheung P.F., Siveke J.T., Wilson I. (2023). Fibroblast Activation Protein-Targeting Minibody-IRDye700DX for Ablation of the Cancer-Associated Fibroblast with Photodynamic Therapy. Cells.

[21] Huang Q.L., Cao X., Chai X., Wang X., Xiao C., Wang J. (2019). The Radiological Imaging Features of Easily Misdiagnosed Epithelioid Glioblastoma in Seven Patients. World Neurosurg..

[22] Omoto K., Matsuda R., Nakagawa I., Motoyama Y., Nakase H. (2018). False-positive inflammatory change mimicking glioblastoma multiforme under 5-aminolevulinic acid-guided surgery: A case report. Surg. Neurol. Int..

[23] Lakhan S.E., Harle L. (2009). Difficult diagnosis of brainstem glioblastoma multiforme in a woman: A case report and review of the literature. J. Med. Case Rep..

[24] Sun H.G., Zu Y. (2015). Aptamers and Their Applications in Nanomedicine. Small.

[25] Vosoughi P., Naghib S.M., Kangarshahi B.M., Mozafari M.R. (2025). A review of RNA nanoparticles for drug/gene/protein delivery in advanced therapies: Current state and future prospects. Int. J. Biol. Macromol..

[26] Zhou G., Latchoumanin O., Hebbard L., Duan W., Liddle C., George J., Qiao L. (2018). Aptamers as targeting ligands and therapeutic molecules for overcoming drug resistance in cancers. Adv. Drug Deliv. Rev..

[27] Xiang D., Zheng C., Zhou S.F., Qiao S., Tran P.H., Pu C., Li Y., Kong L., Kouzani A.Z., Lin J. (2015). Superior Performance of Aptamer in Tumor Penetration over Antibody: Implication of Aptamer-Based Theranostics in Solid Tumors. Theranostics.

[28] Choi J.W., Seo M., Kim K., Kim A.R., Lee H., Kim H.S., Park C.G., Cho S.W., Kang J.H., Joo J. (2023). Aptamer Nanoconstructs Crossing Human Blood-Brain Barrier Discovered via Microphysiological System-Based SELEX Technology. ACS Nano.

[29] Amero P., Khatua S., Rodriguez-Aguayo C., Lopez-Berestein G. (2020). Aptamers: Novel Therapeutics and Potential Role in Neuro-Oncology. Cancers.

[30] de Almeida C.E.B., Alves L.N., Rocha H.F., Cabral-Neto J.B., Missailidis S. (2017). Aptamer delivery of siRNA, radiopharmaceutics and chemotherapy agents in cancer. Int. J. Pharm..

[31] Chen G., Mao D., Wang X., Chen J., Gu C., Huang S., Yang Y., Zhang F., Tan W. (2023). Aptamer-based self-assembled nanomicelle enables efficient and targeted drug delivery. J. Nanobiotechnol..

[32] Xie S., Sun W., Fu T., Liu X., Chen P., Qiu L., Qu F., Tan W. (2023). Aptamer-Based Targeted Delivery of Functional Nucleic Acids. J. Am. Chem. Soc..

[33] Liu W., Zhang K., Zhuang L., Liu J., Zeng W., Shi J., Zhang Z. (2019). Aptamer/photosensitizer hybridized mesoporous MnO_2_ based tumor cell activated ROS regulator for precise photodynamic therapy of breast cancer. Colloids Surf. B Biointerfaces.

[34] Affinito A., Quintavalle C., Esposito C.L., Roscigno G., Giordano C., Nuzzo S., Ricci-Vitiani L., Scognamiglio I., Minic Z., Pallini R. (2020). Targeting Ephrin Receptor Tyrosine Kinase A2 with a Selective Aptamer for Glioblastoma Stem Cells. Mol. Ther. Nucleic Acids.

[35] Affinito A., Quintavalle C., Esposito C.L., Roscigno G., Vilardo C., Nuzzo S., Ricci-Vitiani L., De Luca G., Pallini R., Kichkailo A.S. (2019). The Discovery of RNA Aptamers that Selectively Bind Glioblastoma Stem Cells. Mol. Ther. Nucleic Acids.

[36] Pallini R., Ricci-Vitiani L., Banna G.L., Signore M., Lombardi D., Todaro M., Stassi G., Martini M., Maira G., Larocca L.M. (2008). Cancer stem cell analysis and clinical outcome in patients with glioblastoma multiforme. Clin. Cancer Res..

[37] Esposito C.L., Passaro D., Longobardo I., Condorelli G., Marotta P., Affuso A., de Franciscis V., Cerchia L. (2011). A neutralizing RNA aptamer against EGFR causes selective apoptotic cell death. PLoS ONE.

[38] Palma F., Affinito A., Nuzzo S., Roscigno G., Scognamiglio I., Ingenito F., Martinez L., Franzese M., Zanfardino M., Soricelli A. (2021). miR-34c-3p targets CDK1 a synthetic lethality partner of KRAS in non-small cell lung cancer. Cancer Gene Ther..

[39] Affinito A., Quintavalle C., Chianese R.V., Roscigno G., Fiore D., D’Argenio V., Thomas G., Savarese A., Ingenito F., Cocca L. (2024). MCT4-driven CAF-mediated metabolic reprogramming in breast cancer microenvironment is a vulnerability targetable by miR-425-5p. Cell Death Discov..

[40] Roscigno G., Cirella A., Affinito A., Quintavalle C., Scognamiglio I., Palma F., Ingenito F., Nuzzo S., De Micco F., Cuccuru A. (2020). miR-216a Acts as a Negative Regulator of Breast Cancer by Modulating Stemness Properties and Tumor Microenvironment. Int. J. Mol. Sci..

[41] Pop C.F., Veys I., Bormans A., Larsimont D., Liberale G. (2024). Fluorescence imaging for real-time detection of breast cancer tumors using IV injection of indocyanine green with non-conventional imaging: A systematic review of preclinical and clinical studies of perioperative imaging technologies. Breast Cancer Res. Treat..

[42] Rosenberg A., Fujimura D., Okada R., Furusawa A., Inagaki F., Wakiyama H., Kato T., Choyke P.L., Kobayashi H. (2020). Real-Time Fluorescence Imaging Using Indocyanine Green to Assess Therapeutic Effects of Near-Infrared Photoimmunotherapy in Tumor Model Mice. Mol. Imaging.

[43] Wu M.R., Huang Y.Y., Hsiao J.K. (2019). Use of Indocyanine Green (ICG), a Medical Near Infrared Dye, for Enhanced Fluorescent Imaging-Comparison of Organic Anion Transporting Polypeptide 1B3 (OATP1B3) and Sodium-Taurocholate Cotransporting Polypeptide (NTCP) Reporter Genes. Molecules.

[44] Antaris A.L., Chen H., Cheng K., Sun Y., Hong G.S., Qu C.R., Diao S., Deng Z.X., Hu X.M., Zhang B. (2016). A small-molecule dye for NIR-II imaging. Nat. Mater..

[45] Desmettre T., Devoisselle J.M., Mordon S. (2000). Fluorescence properties and metabolic features of indocyanine green (ICG) as related to angiography. Surv. Ophthalmol..

[46] Lakhin A.V., Tarantul V.Z., Gening L.V. (2013). Aptamers: Problems, solutions and prospects. Acta Naturae.

[47] Yaseen M.A., Yu J., Jung B., Wong M.S., Anvari B. (2009). Biodistribution of encapsulated indocyanine green in healthy mice. Mol. Pharm..

[48] Fisher J.P., Adamson D.C. (2021). Current FDA-Approved Therapies for High-Grade Malignant Gliomas. Biomedicines.

[49] Rodriguez-Camacho A., Flores-Vazquez J.G., Moscardini-Martelli J., Torres-Rios J.A., Olmos-Guzman A., Ortiz-Arce C.S., Cid-Sanchez D.R., Perez S.R., Macias-Gonzalez M.D.S., Hernandez-Sanchez L.C. (2022). Glioblastoma Treatment: State-of-the-Art and Future Perspectives. Int. J. Mol. Sci..

[50] Gao J., Chen Z., Li X., Yang M., Lv J., Li H., Yuan Z. (2022). Chemiluminescence in Combination with Organic Photosensitizers: Beyond the Light Penetration Depth Limit of Photodynamic Therapy. Int. J. Mol. Sci..

[51] Bordoloi B., Goswami A., Roy D., Goswami P., Das I. (2024). Efficacy of Aminolevulinic Acid Mediated Photodynamic Therapy in the Treatment of Oral Premalignant Lesions: A Systematic Review. Asian Pac. J. Cancer Prev..

