# Supplementary material for: Targeting Glioblastoma Stem Cells: A40s Aptamer-NIR-Dye Conjugate for Glioblastoma Visualization and Treatment
Source: Biomolecules. 2025 May 27;15(6):768. doi: 10.3390/biom15060768 (PMC12190191; doi:10.3390/biom15060768)
Supplement: Supplementary file 1 [file biomolecules-15-00768-s001.zip › biomolecules-3521925-submit-images 1.pdf]

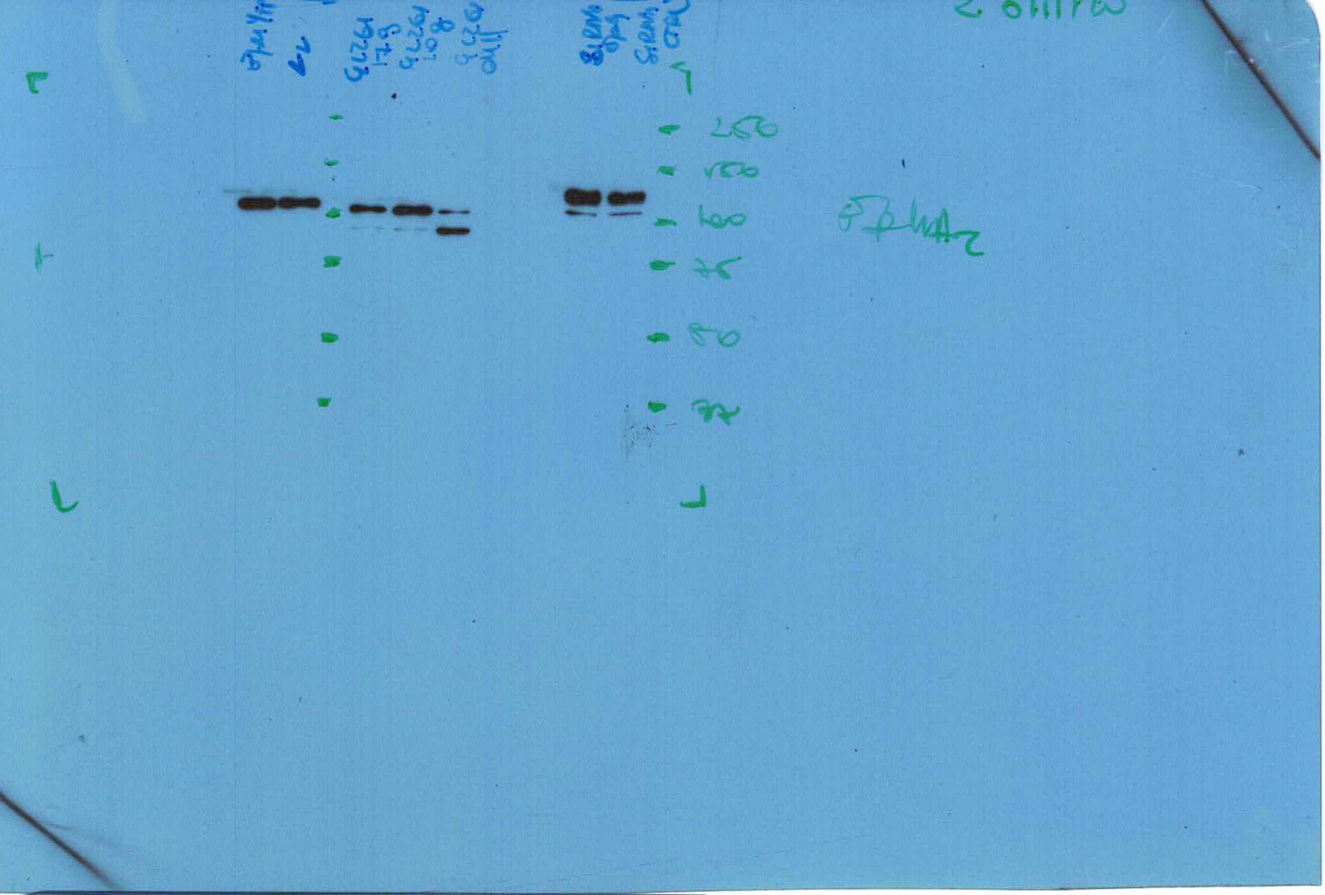

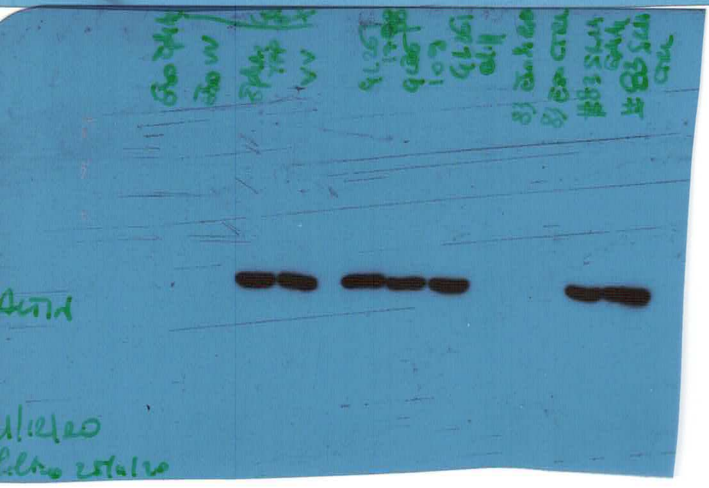
Actin
