# Supplementary material for: Targeting Glioblastoma Stem Cells: A40s Aptamer-NIR-Dye Conjugate for Glioblastoma Visualization and Treatment
Source: Biomolecules. 2025 May 27;15(6):768. doi: 10.3390/biom15060768 (PMC12190191; doi:10.3390/biom15060768)
Supplement: Supplementary file 1 [file biomolecules-15-00768-s001.zip › biomolecules-3521925-supplementary.pdf]

A)

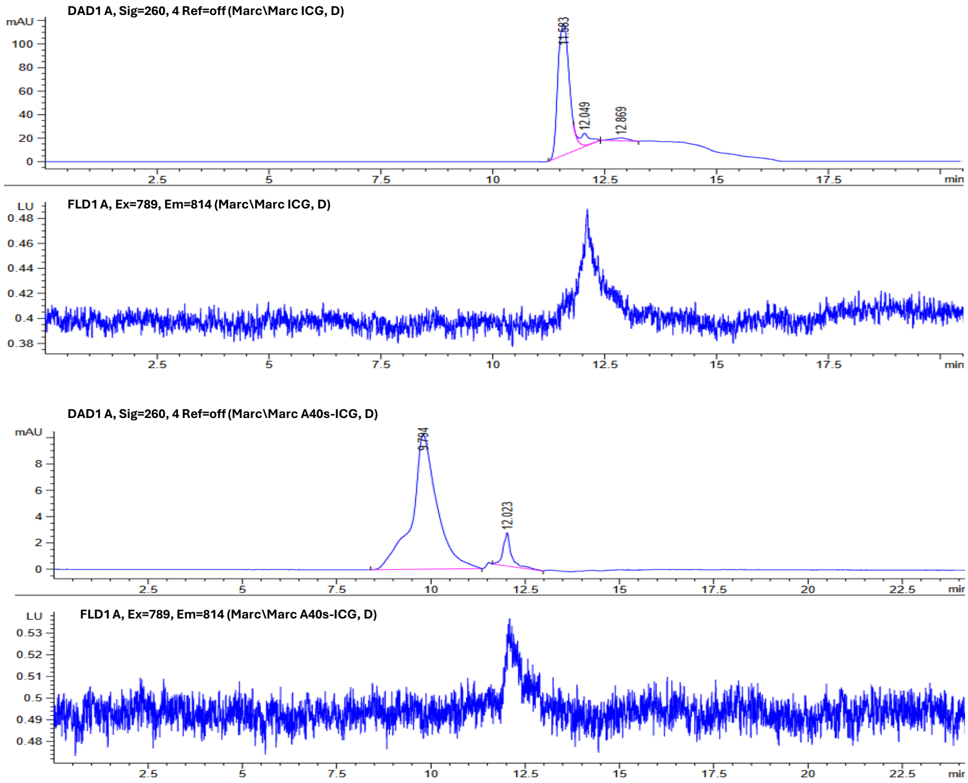

B)

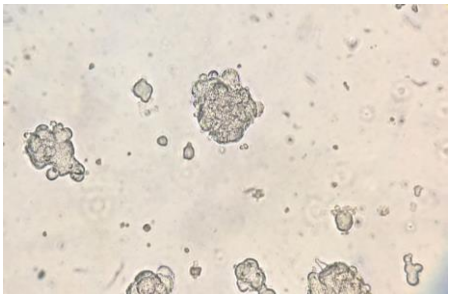

Gl261 stem-like

C)

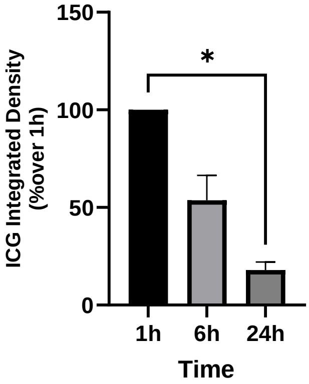

D)

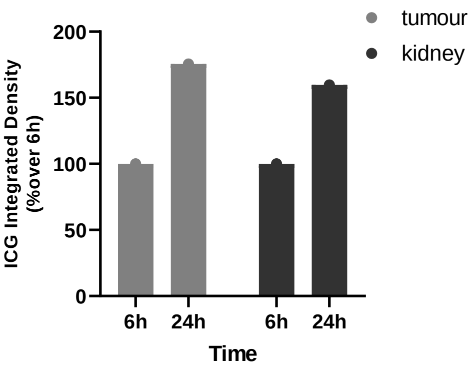

Figure legend

Supplemental Figure S1. A40s-ICG qualitative and in vivo quantitative evaluation **(A)** Fluorescence-detection size exclusion high-performance liquid chromatography (FSE-HPLC) spectra for ICG (up panel) and A40s-ICG (bottom panel) (DAD= 260nm and FLD= Ex/Em 789/814nm). **(B)** GL261-luc2 cells cultured as stem-like (suspension, 3D culture) in B27, EGF and FGF supplemented medium (5x magnification). **(C)** Integrated Density analysis of the ICG-mediated fluorescent signal of figure 4 quantified by ImageJ after 1-6 and 24 hours. **(D)** Integrated Density analysis of the ICG-mediated fluorescent signal of tumor and kidneys in figure 5, quantified by ImageJ at sacrifice time- 6 and 24h.
